# Distribution characteristics and influencing factors of homocyteine in an apparently healthy examined population

**DOI:** 10.1186/s12872-021-02238-5

**Published:** 2021-09-10

**Authors:** Fang Bao, Ming Cui, Xiuying Shi, Shaoqing Ju, Hui Cong

**Affiliations:** 1 Department of Laboratory Medicine, Affiliated Hospital of Nantong University, Nantong, 226001 China; 2 Department of Blood Transfusion, Affiliated Hospital of Nantong University, Nantong, 226001 China

**Keywords:** Homocysteine, Creatinine, Uric acid, High-density lipoprotein cholesterol

## Abstract

**Background:**

Homocysteine (Hcy) is considered to be a risk factor for cardiovascular and cerebrovascular diseases. Few studies have evaluated the distribution of Hcy on a large-scale health examination. Accordingly, this study aimed to investigate the level and distribution of Hcy in the population with healthy physical examination and the correlation with other biomarkers, and analyzed for cardiovascular and other diseases.

**Methods:**

Measurements of serum Hcy, TC, TG, LDL-c, HDL-c, ALT, ALP, γ-GT, TBIL, GLU, urea, Cr, UA, and related metabolic risk factors were selected for analysis from 8063 medical examination samples collected from February 2017 to April 2020. The relationship between Hcy and other biochemical indicators were evaluated with the multivariate regression model of age, gender, smoking, drinking, body mass index (BMI), systolic blood pressure (SBP), and diastolic blood pressure (DBP).

**Results:**

Among 8063 cases, the age, BMI, SBP, and DBP of the high-Hcy group were higher than those of the low-Hcy group, the difference was statistically significant (*P* < 0.001), and the proportion of males, smoking, and drinking were higher than the low-Hcy group, the difference was statistically significant (*P* < 0.001); Hcy of the abnormal GLU group is higher than the normal GLU group (*P* = 0.002) and the Hcy of abnormal TG and HDL is higher than that of the normal blood lipid group (*P* < 0.001); Hcy of people with abnormal UA and Urea was higher than that of people with normal renal function (*P* < 0.001, *P* = 0.007). In multivariate analysis, lnHDL-C was negatively correlated with lnHcy (β = − 0.038, SE = 0.016, *P* = 0.019), lnCr was positively correlated with lnHcy (β = 0.055, SE = 0.016, *P* < 0.001), lnUA and lnHcy were positive correlated (β = 0.043, SE = 0.019, *P* = 0.022).

**Conclusion:**

Hcy is closely related to HDL-c, Cr, and UA, which indicates that Hcy may affect the metabolism of HDL-c and UA, and can also be used as an auxiliary diagnostic index for kidney injury.

## Background

Homocysteine (Hcy) is a sulfur-containing amino acid produced during the metabolism of methionine in cells of the body which involves folic acid and vitamin B12. Its pathogenic mechanism includes vascular endothelial damage, stimulating smooth muscle cell proliferation, affecting coagulation and thrombus activation, etc. The elevation of Hcy is considered an independent risk factor for cardiovascular and cerebrovascular diseases [1, 2]. In this paper, we report the level and distribution characteristics of Hcy and their correlation between other biochemical indicators by analyzing a population of 8063 medical examination results, as well as the possible influencing factor for cardiovascular and cerebrovascular diseases.

## Methods

The subjects of this study came from a total of 8063 cases of the physical examination population in our hospital from February 2017 to April 2020. Inclusion criteria: (1) Daily physical fitness; (2) No acute or chronic infections, tumors or rheumatic diseases were found after clinical examination. Exclusion criteria: (1) Recent use of drugs affecting blood Hcy level and blood lipid level; (2) Subjects with severe liver and kidney insufficiency, anemia, and cardiac insufficiency; (3) Genetic metabolic diseases, malignant tumors, connective tissue diseases, etc. This research protocol was approved by the Ethics Committee of Nantong University Affiliated Hospital. The distribution characteristics of Hcy, blood lipids and other biochemical indicators, systolic blood pressure (SBP), diastolic blood pressure (DBP), body mass index (BMI), and pulse of the physical examination population were observed. The subjects were stratified according to gender and Hcy in low (Hcy ≤ 15 μmol/L) and high (Hcy > 15 μmol/L) Hcy group. Other indicators are divided into normal group and abnormal group according to the literature [3–10].

5 mL of venous blood was collected from subjects fasted for 12 h into a vacuum test tube containing separation gel. After the blood coagulated, the serum was separated by centrifugation at 2062*g* for 10 min within 2 h and tested on the machine. All tests were carried out with the internal quality control when the instrument and reagents were in normal condition, and were carried out in strict accordance with the reagent and instrument standard operating procedures (SOP). All biochemical indexes measured on Beckman-Coulter AU5800 automatic analyzer. Hcy (enzyme cycling method) kit (LOT: 0200103) and calibrator were purchased from Qiangsheng Biotechnology Co., Ltd., quality control products were provided by Shanghai Kunlai Biotechnology Company; ALT (lactate dehydrogenase method), ALP (NPP substrate-AMP buffer method)), γ-GT (rate method), TBIL (diazonium method), TG (GPO-POD method), TC (cholesterol oxidase method), LDL-c (direct method), HDL-c (direct method), GLU (hexokinase method), BUN (urease-glutamate dehydrogenase method), Cr (sarcosine oxidase method), UA (uricase-peroxidase method) are tested by Beckman-Coulter Original kits and calibrators, quality control products are provided by Bio-Rad.

### Statistical analysis

Statistical software stata 20.0 was used for data analysis, skewness and kurtosis normality test (sktest). Numerical variables of the normal distribution were expressed by the mean ± standard deviation, and the comparison between two groups was performed by the T test; the median (interquartile range) was used to express numerical variables of the non-normal distribution, and the Mann–Whitney U test was used for comparison between the two groups. Kruskal–Wallis test was used for comparison among multiple groups. The qualitative data were all expressed by the number of cases (percentage), and the chi-square test was used for comparison between groups. This study analyzed the relationship between Hcy and TC, TG, LDL-c, HDL-c, ALT, ALP, TBIL, γ-GT, Urea, Cr and UA through single factor, multivariate linear regression and logistic regression models. The skewed numerical variables were analyzed after natural logarithmic transformation, and the multivariate regression was adjusted for age, gender, smoking, drinking, pulse, and BMI. *P* < 0.05 was considered statistically significant.

## Results

### General characteristics

This study included 8063 subjects with an average age of (50.88 ± 11.92) years old, BMI (25.02 ± 3.39) kg/m^2^, SBP (130.54 ± 18.85) mmHg, DBP (78.66 ± 11.96) mmHg, pulse (77.54 ± 11.31) times/min. Among them, 5478 cases were male, accounting for 67.94%, and 2585 cases were female, accounting for 32.06%, All subjects were aged between 20 and 79 years old. The BMI, SBP, DBP, and glucose of men were higher than women, and the difference was statistically significant (*P* < 0.001), while the pulse rate of men was lower than that of women with a statistically significant difference (*P* < 0.001). Among men, the proportion of smoking was 23.95% and the proportion of drinking was 64.8%, which were much higher than those of women. Male ALT, γ-GT, TBIL, TG, LDL-c, Urea, Cr, UA were all higher than females, and the difference was statistically significant (*P* < 0.001). TC, HDL-C were lower than females (*P* < 0.001), and there was no statistical difference in ALP between the two groups (*P* = 0.054), Table 1.

**Table 1:** Distribution characteristics of the study population grouped by gender

	Total (n = 8063)	Male (n = 5478)	Female (n = 2585)	*P*
Age (year-old), mean ± SD	50.88 ± 11.92	49.98 ± 11.49	52.87 ± 12.59	< 0.001
BMI (kg/m^2^), mean ± SD	25.02 ± 3.39	25.54 ± 3.22	23.91 ± 3.48	< 0.001
SBP (mmHg), mean ± SD	130.54 ± 18.85	131.49 ± 17.28	128.52 ± 21.68	< 0.001
DBP (mmHg), mean ± SD	78.66 ± 11.96	81.13 ± 11.46	73.43 ± 11.30	< 0.001
Pulse (bpm), mean ± SD	77.54 ± 11.31	76.77 ± 11.18	79.19 ± 11.42	< 0.001
Smoking status, N (%)				< 0.001
Current and former	1312 (16.27)	1312 (23.95)	0 (0.00)	
Never	6751 (83.73)	4166 (76.05)	2585 (100)	
Alcohol dringking, N (%)				< 0.001
Current and former	1930 (23.94)	1928 (35.20)	2 (0.08)	
Never	6133 (76.06)	3550 (64.80)	2583 (99.92)	
Hcy (μmol/L), M_(P25–P75)_	10.2 (8.3–12.8)	10.9 (9–13.8)	8.6 (7.1–10.7)	< 0.001
ALT (U/L), M_(P25–P75)_	24 (18–34)	26.5 (20–38)	19 (15–26)	< 0.001
ALP (U/L), M_(P25–P75)_	81 (67–96)	81 (69–95)	80 (65–98)	0.054
γ-GT (U/L), M_(P25–P75)_	28 (19–45)	33 (23–53)	18 (14–27)	< 0.001
TBIL (μmol/L), M_(P25–P75)_	13.7 (10.8–17.3)	14.4 (11.6–18.1)	12 (9.6–15.1)	< 0.001
GLU (mmol/L), M_(P25–P75)_	5.3 (4.9–5.8)	5.3 (5–5.8)	5.2 (4.9–5.6)	< 0.001
TC (mmol/L), M_(P25–P75)_	5.1 (4.5–5.8)	5.1 (4.5–5.7)	5.2 (4.6–5.8)	< 0.001
TG (mmol/L), M_(P25–P75)_	1.4 (0.95–2.1)	1.54 (1.05–2.32)	1.14 (0. 81–1.67)	< 0.001
HDL-c (mmol/L), M_(P25–P75)_	1.27 (1.09–1.49)	1.2 (1.05–1.39)	1.44 (1.24–1.67)	< 0.001
LDL-c (mmol/L), M_(P25–P75)_	3.06 (2.54–3.57)	3.07 (2.56–3.59)	3.01 (2.51–3.53)	0.013
Urea (mmol/L), M_(P25–P75)_	5.3 (4.5–6.1)	5.4 (4.6–6.3)	5 (4.1–5.9)	< 0.001
Cr (μmol/L), M_(P25–P75)_	67 (57–76)	72 (66–80)	53 (48–59)	< 0.001
UA (μmol/L), M_(P25–P75)_	332 (269–393)	363 (313–416)	256 (218–304)	< 0.001

### Comparison of Hcy between different groups

Table 2 shows the comparison of Hcy among different groups. Hcy of males are higher than that of females (*P* < 0.001), Hcy of smokers and drinkers are higher than the control group (*P* < 0.001), Hcy of the overweight and obese groups are higher than the thin and normal BMI groups. (*P* < 0.001), Hcy of the abnormal blood pressure group is higher than the normal hypertension group (*P* < 0.001), Hcy of the abnormal GLU group is higher than the normal GLU group (*P* = 0.002) and the Hcy of abnormal TG and HDL is higher than that of the normal blood lipid group (*P* < 0.001); Hcy of people with abnormal UA and Urea was higher than that of people with normal renal function (*P* < 0.001, *P* = 0.007).

**Table 2:** Comparison of Hcy between different groups

	Hcy (n = 8063)	Z/H	P	Male			Female		
				Hcy(n = 5478)	Z/H	P	Hcy(n = 2585)	Z/H	P
*Sex*		30.910	< 0.001		–	–		–	–
Male	10.9 (9.0–13.8)			–			–		
Female	8.6 (7.1–10.7)			–			–		
*Smoking status*		− 8.896	< 0.001		0.581	0.5610		–	–
Current and former	10.9 (9.0–13.8)			10.9 (9.0–13.8)			–		
Never	10.1 (8.1–12.7)			11.0 (9.1–13.8)			8.6 (7.1–10.7)		
*Alcohol drinking*		− 11.048	< 0.001		1.183	0.2368		0.660	0.510
Current and former	10.8 (9.0–13.6)			10.8 (9.0–13.6)			7.8 (6.9–8.7)		
Never	10.0 (8.1–12.6)			11.0 (9.1–13.9)			8.6 (7.1–10.7)		
*BMI(kg/m*^*2*^*)*		117.157	< 0.001		4.565	0.2066		32.569	< 0.001
Weight loss	9.2 (7.9–12.4)			11.8 (8.9–15.9)			8.6 (7.2–10.2)		
Normal	9.7 (7.9–12.2)			10.7 (8.9–13.6)			8.4 (7.0–10.4)		
Overweight	10.5 (8.5–13.2)			11.1 (9.1–13.8)			8.7 (7.1–10.9)		
Obesity	10.6 (8.7–13.5)			10.8 (9–13.95)			9.5 (7.9–11.5)		
*Blood pressure (mmHg)*		− 12.666	< 0.001		− 4.511	< 0.0001		− 9.976	< 0.001
Normal	9.6 (7.8–12.2)			10.7 (8.8–13.4)			8.2 (6.8–10.0)		
Abnormal	10.6 (8.7–13.4)			11.1 (9.2–14.1)			9.2 (7.7–11.4)		
*ALT (U/L)*		− 0.004	0.997		2.069	0.0386		0.582	0.560
Normal	10.2 (8.3–12.8)			11.0 (9.1–13.9)			8.6 (7.2–10.7)		
Abnormal	10.3 (8.3–12.7)			10.7 (8.8–13.3)			8.5 (6.9–10.9)		
*ALP (U/L)*		2.573	0.010		1.676	0.0938		0.349	0.728
Normal	10.3 (8.3–12.8)			11.0 (9.0–13.8)			8.6 (7.2–10.7)		
Abnormal	9.6 (8.0–12.4)			10.5 (8.8–13.7)			8.55 (7.0–10.9)		
*γ-GT (U/L)*		− 5.507	< 0.001		− 1.082	0.2792		− 1.246	0.213
Normal	10.1 (8.2–12.7)			10.9 (9–13.7)			8.6 (7.1–10.6)		
Abnormal	10.8 (8.6–13.5)			11.1 (9–14.1)			8.7 (7.3–11.3)		
*TBIL (μmol/L)*		− 7.236	< 0.001		− 4.158	< 0.0001		− 0.749	0.454
Normal	10.1 (8.2–12.7)			10.9 (9.0–13.7)			8.6 (7.2–10.7)		
Abnormal	11.2 (9.1–13.9)			11.7 (9.6–14.2)			8.8 (7.0–11.6)		
*GLU (mmol/L)*		− 3.109	0.002		2.112	0.0347		− 3.922	< 0.001
Normal	10.2 (8.2–12.8)			11.0 (9–13.8)			8.6 (7.1–10.6)		
Abnormal	10.5 (8.6–13.1)			10.8 (9–13.7)			9.2 (7.6–11.4)		
*TC (mmol/L)*		− 1.192	0.233		− 1.222	0.2216		− 3.506	< 0.001
Normal	10.2 (8.2–12.8)			10.9 (9.0–13.8)			8.5 (7.0–10.4)		
Abnormal	10.2 (8.3–12.9)			11.1 (9.1–13.8)			8.8 (7.3–11.0)		
*TG (mmol/L)*		− 9.402	< 0.001		− 2.261	0.0237		− 4.309	< 0.001
Normal	10.0 (8.0–12.5)			10.9 (9.0–13.6)			8.5 (7.0–10.6)		
Abnormal	10.6 (8.7–13.4)			11.0 (9.1–14.0)			9.0 (7.5–11.1)		
*HDL (mmol/L)*		− 5.308	< 0.001		0.698	0.4855		0.0274	0.027
Normal	10.1 (8.2–12.7)			10.9 (9.0–13.9)			8.6 (7.1–10.7)		
Abnormal	10.7 (8.7–13.4)			10.9 (8.9–13.5)			8.8 (7.7–12.1)		
*LDL (mmol/L)*		− 0.313	0.754		1.086	0.2776		− 0.879	0.380
Normal	10.2 (8.3–12.8)			11.0 (9.0–13.8)			8.6 (7.1–10.6)		
Abnormal	10.2 (8.3–12.8)			10.8 (9.0–13.7)			8.7 (7.2–10.9)		
*Urea (mmol/L)*		− 2.691	0.007		− 1.822	0.068		− 1.759	0.079
Normal	10.2 (8.3–12.8)			10.9 (9.0–13.8)			8.6 (7.1–10.6)		
Abnormal	10.9 (8.3–14.1)			11.6 (9.2–14.8)			9.4 (7.2–11.6)		
*Cr(μmol/L)*		0.030	0.976		− 0.188	0.851		1.649	0.099
Normal	10.2 (8.3–12.8)			10.9 (9.0–13.8)			8.7 (7.2–10.7)		
Abnormal	10.2 (8.0–13.4)			11.0 (8.8–14.2)			8.2 (6.7–10.8)		
*UA(μmol/L)*		− 13.132	< 0.001		− 5.409	< 0.001		− .389	< 0.001
Normal	10.0 (8.1–12.5)			10.8 (8.9–13.6)			8.5 (7.0–10.5)		
Abnormal	11.1 (9.2–14.2)			11.3 (9.4–14.5)			9.8 (8.4–12.5)		

A history of smoking or drinking was defined as abnormal smoking or drinking

Regardless of gender, Hcy was higher in the group with abnormal TG、UA and BP than in the group with normal (*P* < 0.001); According to gender stratification, the Hcy of male abnormal GLU group was lower than that of normal GLU group (*P* = 0.035), and the Hcy of female abnormal GLU group was higher than that of normal GLU group (*P* < 0.001). In addition, Hcy of men with abnormal ALT was lower than that of normal ALT (*P* = 0.039), and Hcy of men with abnormal TBIL was higher than that of normal TBIL (*P* < 0.001); Hcy in overweight and obese women was higher than that in lean and normal BMI groups (*P* < 0.001), and Hcy in group with abnormal TC and HDL was higher than that in normal population (*P* < 0.001, 0.027).

### Comparison of high and low Hcy indicators by gender

Among men, the age, SBP, DBP, Cr and UA of the high-Hcy group were higher than those of the low-Hcy group, *P* < 0.001. The GLU, between the high-Hcy group was lower than that of the low-Hcy group, *P* < 0.001; the γ-GT, TBIL and TG of the high-Hcy group were higher than those of the low-Hcy group (*P* = 0.023, < 0.001, 0.011).

Among women, the age, BMI, SBP, and DBP of the high-Hcy group were significantly higher than those of the low-Hcy group. The ALP of the high-Hcy group was higher than that of the low-Hcy group, *P* = 0.001. The TG of the high-Hcy group was significantly higher than that of the low-Hcy group, *P* = 0.003, while the HDL-C was lower than the low-Hcy group, *P* = 0.005; Both Cr and UA were significantly higher than the low-Hcy group, *P* < 0.001. See Table 3 for details.

**Table 3:** The characteristics of the population in the high and low Hcy groups are stratified by gender

	Male			Female		
	Low Hcy (n = 4479)	High Hcy (n = 999)	*P*	Low Hcy (n = 2479)	High Hcy (n = 106)	*P*
Age (year-old), mean ± SD	49.66 ± 11.16	51.37 ± 12.78	< 0.001	52.42 ± 12.50	61.79 ± 11.25	< 0.001
BMI (kg/m^2^), mean ± SD	25.54 ± 3.21	25.55 ± 3.28	0.941	23.86 ± 3.40	25.14 ± 4.88	< 0.001
SBP (mmHg), mean ± SD	130.92 ± 16.97	134.05 ± 18.36	< 0.001	128.04 ± 21.59	139.77 ± 21.00	< 0.001
DBP (mmHg), mean ± SD	80.90 ± 11.34	82.16 ± 11.96	0.002	73.27 ± 11.24	77.00 ± 12.03	< 0.001
Pulse (bpm), mean ± SD	76.71 ± 11.13	77.04 ± 11.41	0.395	79.27 ± 11.43	77.32 ± 11.00	0.085
ALT (U/L), M_(P25–P75)_	26 (20–38)	27 (19–38)	0. 950	19 (15–26)	20 (14–25)	0.715
ALP (U/L), M_(P25–P75)_	80 (68–95)	82 (69–97)	0.056	80 (64–97)	86.5 (71–108)	0.001
γ-GT (U/L), M_(P25–P75)_	33 (23–52)	34 (23–56)	0.023	18 (14–27)	20 (15–30)	0.058
TBIL (μmol/L), M_(P25–P75)_	14.3 (11.5–18)	15.1 (12.1–18.8)	< 0.001	12 (9.6–15.1)	12.6 (10.6–15. 5)	0.059
GLU (mmol/L), M_(P25–P75)_	5.3 (5–5.9)	5.2 (4.9–5.7)	< 0.001	5.2 (4.9–5.6)	5.3 (4.9–6.2)	0.011
TC (mmol/L), M_(P25–P75)_	5.1 (4.5–5.7)	5.1 (4.5–5.8)	0.652	5.2 (4.6–5.8)	5.3 (4.6–5.9)	0.395
TG (mmol/L), M_(P25–P75)_	1.53 (1.03–2.29)	1.6 (1.11–2.48)	0.011	1.13 (0.8–1.66)	1.36 (0.97–2)	0.003
HDL-c (mmol/L), M_(P25–P75)_	1.2 (1.04–1.4)	1.2 (1.06–1.37)	0.775	1.44 (1.24–1.67)	1.37 (1.19–1.55)	0.005
LDL-c (mmol/L), M_(P25–P75)_	3.08 (2.57–3.6)	3.06 (2.52–3.58)	0.197	3.01 (2.51–3.53)	2.99 (2.47–3.55)	0.594
Urea (mmol/L), M_(P25–P75)_	5.4 (4.6–6.2)	5.4 (4.6–6.3)	0.228	4.9 (4.1–5.8)	5.3 (4.4–6.3)	0.006
Cr (μmol/L), M_(P25–P75)_	72 (65–79)	75 (68–83)	< 0.001	53 (48–59)	57.5 (52–66)	< 0.001
UA (μmol/L), M_(P25–P75)_	361 (312–413)	370 (324–429)	< 0.001	255 (218–301)	290 (235–377)	< 0.001

Define low Hcy group (Hcy ≤ 15 μmol/L) and high Hcy group (Hcy > 15 μmol/L)

### Linear regression model analysis of the effect of serum Hcy level on blood lipid level

As shown in Table 4, the single factor linear regression model analysis results show that lnHcy is negatively correlated with lnHDL-c, and lnHcy is positively correlated with lnTG, lnALT, lnALP, lnγ-GT, lnTBIL, lnUrea, lnCr, and lnUA. The adjusted multivariate linear regression model analysis of gender, age, BMI, smoking and drinking showed that lnTG was positively correlated with lnHcy (β = 0.080, SE = 0.021, *P* < 0.001), and lnHDL-c was negatively correlated with lnHcy (β = − 0.021, SE = 0.008, *P* = 0.011), lnALT was negatively correlated with lnHcy (β = − 0.053, SE = 0.018, *P* = 0.003), and lnTBIL was positively correlated with lnHcy (β = 0.054, SE = 0.014, *P* < 0.001), lnCr was positively correlated with lnHcy (β = 0.065, SE = 0.006, *P* < 0.001), and lnUA was positively correlated with lnHcy (β = 0.069, SE = 0.009, *P* < 0.001). For the abnormal Hcy group (Hcy > 15 μmol/L), in univariate analysis, lnTC was negatively correlated with lnHcy (β = − 0.032, SE = 0.016, *P* = 0.046), and lnHDL-c was negatively correlated with lnHcy (β = − 0.060, SE = 0.017, *P* = 0.001), lnALP is positively correlated with lnHcy (β = 0.062, SE = 0.022, *P* = 0.004), and lnUrea is negatively correlated with lnHcy (β = − 0.045, SE = 0.021, *P* = 0.034), LnCr was positively correlated with lnHcy (β = 0.065, SE = 0.016, *P* < 0.001), and lnUA was positively correlated with lnHcy (β = 0.062, SE = 0.020, *P* = 0.002). In multivariate analysis, lnTC was negatively correlated with lnHcy (β = − 0.036, SE = 0.016, *P* = 0.027), lnHDL-C was negatively correlated with lnHcy (β = − 0.038, SE = 0.016, *P* = 0.019), lnALP It is positively correlated with lnHcy (β = 0.068, SE = 0.022, *P* = 0.002), lnCr is positively correlated with lnHcy (β = 0.055, SE = 0.016, *P* < 0.001), lnUA and lnHcy were positively correlated (β = 0.043, SE = 0.019, *P* = 0.022). See Table 4 for details.

**Table 4:** Analysis of linear regression model of serum Hcy on each index

	Single factor analysis		Multiple-factor analysis	
	β(SE)	*P*	β(SE)	*P*
*LnTC*				
lnHcy	0.005 (0.007)	0.458	0.009 (0.007)	0.216
Hcy (vs. Hcy ≤ 15 μmol/L)	− 0.032 (0.016)	0.046	− 0.036 (0.016)	0.027
*LnTG*				
lnHcy	0.212 (0.022)	< 0.001	0.080 (0.021)	< 0.001
Hcy (vs. Hcy ≤ 15 μmol/L)	0.090 (0.049)	0.068	0.047 (0.047)	0.324
*LnHDL-c*				
lnHcy	− 0.087 (0.009)	< 0.001	− 0.021 (0.008)	0.011
Hcy (vs. Hcy ≤ 15 μmol/L)	− 0.060 (0.017)	0.001	− 0.038 (0.016)	0.019
*LnLDL-c*				
lnHcy	− 0.004 (0.010)	0.661	− 0.014 (0.010)	0.169
Hcy (vs. Hcy ≤ 15 μmol/L)	− 0.021 (0.023)	0.365	− 0.034 (0.023)	0.140
*LnALT*				
lnHcy	0.079 (0.019)	< 0.001	− 0.053 (0.018)	0.003
Hcy (vs. Hcy ≤ 15 μmol/L)	0.037 (0.043)	0.389	− 0.020 (0.041)	0.621
*LnALP*				
lnHcy	0.037 (0.010)	< 0.001	0.008 (0.011)	0.401
Hcy (vs. Hcy ≤ 15 μmol/L)	0.062 (0.022)	0.004	0.068 (0.022)	0.002
*Lnγ-GT*				
lnHcy	0.206 (0.025)	< 0.001	− 0.031 (0.023)	0.168
Hcy (vs. Hcy ≤ 15 μmol/L)	0.072 (0.057)	0.210	0.036 (0.054)	0.503
*LnTBIL*				
lnHcy	0.122 (0.013)	< 0.001	0.054 (0.014)	< 0.001
Hcy (vs. Hcy ≤ 15 μmol/L)	0.014 (0.030)	0.633	0.020 (0.030)	0.506
*LnUrea*				
lnHcy	0.070 (0.009)	< 0.001	0.017 (0.009)	0.058
Hcy (vs. Hcy ≤ 15 μmol/L)	− 0.045 (0.021)	0.034	− 0.030 (0.021)	0.159
*lnCr*				
lnHcy	0.177 (0.008)	< 0.001	0.065 (0.006)	< 0.001
Hcy (vs. Hcy ≤ 15 μmol/L)	0.065 (0.016)	< 0.001	0.055 (0.016)	< 0.001
*lnUA*				
lnHcy	0.196 (0.010)	< 0.001	0.069 (0.009)	< 0.001
Hcy (vs. Hcy ≤ 15 μmol/L)	0.062 (0.020)	0.002	0.043 (0.019)	0.022

Multivariate analysis adjusted for age, gender, smoking, drinking, and BMI. LnHcy is the logarithm of Hcy, which is a continuous variable, Hcy is a binary variable, vs. refers to the comparison group, high Hcy > 15 μmol/L vs. low Hcy ≤ 15 μmol/L

### Logistic regression model analysis of serum Hcy on each index

The single factor logistic regression model showed that high lnHcy is the occurrence of high TG (OR 1.870, 95% CI 1.581–2.212, *P* < 0.001), low HDL-C (OR 1.803, 95% CI 1.404–2.316, *P* < 0.001), abnormal γ-GT (OR 1.270, 95% CI 1.028–1.569, *P* = 0.027), high TBIL (OR 2.456, 95% CI 1.741–3.464, *P* < 0.001), high UA (OR 3.106, 95% CI 2.439–3.956, *P* < 0.001) risk factors. High lnHcy is a protective factor for abnormal ALP (OR 0.692, 95% CI 0.531–0.900, *P* = 0.006) and abnormal Cr (OR 0.737, 95% CI 0.565–0.960, *P* = 0.023); multivariate logistic regression model analysis results show that high lnHcy is a risk factor for high TG (OR 1.281, 95% CI 1.078–1.523, *P* = 0.005), high UA (OR 2.008, 95% CI 1.565–2.575, *P* < 0.001) and abnormal TBIL (OR 1.707, 95% CI 1.205–2.418, *P* = 0.003). High lnHcy is a protective factor for abnormal Cr (OR 0.663, 95% CI 0.508–0.866, *P* = 0.003) and high LDL-c (OR 0.820, 95% CI 0.699–0.962, *P* = 0.015). For the abnormal Hcy group (Hcy > 15 μmol/L), single-factor logistic regression showed that high lnHcy is low HDL-C (OR 1.772, 95% CI 1.184–2.653, *P* = 0.005), abnormal ALP (OR 1.940, 95% CI 1.093–3.444, *P* = 0.024), high UA (OR 1.485, 95% CI 1.052–2.096, *P* = 0.024), abnormal Cr (OR 2.086, 95% CI 1.271–3.366, *P* = 0.003) risk factors; multivariate logistic regression analysis results show that high lnHcy is a risk factor for low HDL-C (OR 1.558, 95% CI 1.017–2.386, *P* = 0.042), abnormal ALP (OR 1.992, 95% CI 1.111–3.571, *P* = 0.021), high UA (OR 1.487, 95% CI 1.037–2.131, *P* = 0.031) and abnormal Cr (OR 2.241, 95% CI 1.364–3.681, *P* = 0.001). See Table 5 for details.

**Table 5:** Logistic regression model analysis of serum Hcy on each index

	Single factor analysis		Multiple-factor analysis	
	OR (95%CI)	*P*	OR (95%CI)	*P*
*TG* < *1.7 mmol/L*				
lnHcy	1.870 (1.581–2.212)	< 0.001	1.281 (1.078–1.523)	0.005
Hcy (vs. Hcy ≤ 15 μmol/L)	1.285 (0.928–1.780)	0.131	1.116 (0.791–1.573)	0.533
*TC* < *5.2 mmol/L*				
lnHcy	1.054 (0.911–1.219)	0.479	1.083 (0.929–1.263)	0.307
Hcy (vs. Hcy ≤ 15 μmol/L)	0.795 (0.573–1.104)	0.171	0.743 (0.531–1.041)	0.084
*HDL-C* > *1.0 mmol/L*				
lnHcy	1.803 (1.404–2.316)	< 0.001	1.065 (0.835–1.359)	0.613
Hcy (vs. Hcy ≤ 15 μmol/L)	1.772 (1.184–2.653)	0.005	1.558 (1.017–2.386)	0.042
*LDL-c* < *3.4 mmol/L*				
lnHcy	0.905 (0.776–1.055)	0.201	0.820 (0.699–0.962)	0.015
Hcy (vs. Hcy ≤ 15 μmol/L)	0.859 (0.603–1.225)	0.401	0.819 (0.570–1.177)	0.280
*γ-GT (10–60 U/L (7–45 U/L))*				
lnHcy	1.270 (1.028–1.569)	0.027	0.902 (0.729–1.116)	0.343
Hcy (vs. Hcy ≤ 15 μmol/L)	1.186 (0.808–1.740)	0.384	1.108 (0.744–1.649)	0.615
*ALT (9–50 U/L (7–40 U/L))*				
lnHcy	0.899 (0.720–1.123)	0.348	0.791 (0.627–0.998)	0.048
Hcy (vs. Hcy ≤ 15 μmol/L)	1.411 (0.880–2.260)	0.153	1.175 (0.717–1.924)	0.522
*ALP (45–125 U/L (35–100 U/L))*				
lnHcy	0.692 (0.531–0.900)	0.006	0.726 (0.550–0.958)	0.024
Hcy (vs. Hcy ≤ 15 μmol/L)	1.940 (1.093–3.444)	0.024	1.992 (1.111–3.571)	0.021
*TBIL* ≤ *23 μmol/L*				
lnHcy	2.456 (1.741–3.464)	< 0.001	1.707 (1.205–2.418)	0.003
Hcy (vs. Hcy ≤ 15 μmol/L)	1.211 (0.736–1.993)	0.452	1.241 (0.750–2.055)	0.401
*Urea (3.1–8.8 mmol/L)*				
lnHcy	1.181 (0.767–1.817)	0.450	1.046 (0.675–1.623)	0.839
Hcy (vs. Hcy ≤ 15 μmol/L)	0.817 (0.367–1.818)	0.620	0.963 (0.433–2.145)	0.927
*UA (UA* ≤ *420 umol/L(UA* ≤ *360umol/L))*				
lnHcy	3.106 (2.439–3.956)	< 0.001	2.008 (1.565–2.575)	< 0.001
Hcy (vs. Hcy ≤ 15 μmol/L)	1.485 (1.052–2.096)	0.024	1.487 (1.037–2.131)	0.031
*Cr (57–97 μmoI/L (41–73 μmoI/L))*				
lnHcy	0.737 (0.565–0.960)	0.023	0.663 (0.508–0.866)	0.003
Hcy (vs. Hcy ≤ 15 μmol/L)	2.068 (1.271–3.366)	0.003	2.241 (1.364–3.681)	0.001

Multivariate analysis adjusted for age, gender, smoking, drinking, and BMI. LnHcy is the logarithm of Hcy, which is a continuous variable, Hcy is a binary variable, vs. refers to the comparison group, high Hcy > 15 μmol/L vs. low Hcy ≤ 15 μmol/L. The normal reference value ranges of TBIL, Urea, ALP and Cr are indicated by brackets. If there are double brackets, the outer brackets indicate the normal reference value range for men, and the inner brackets indicate the normal reference value range for women. The normal range of Cr and Urea in parentheses indicates the 20–59 years old population, the 60–79 years old population Cr normal range is (57–111 μmoI/L (41–81 μmoI/L)); the 60–79 year old population Urea's normal range is (3.6–9.5 mmol/L (3.1–8.8 mmol/L))

## Discussion

The baseline data collected in this study showed that the smoking and drinking proportion of men was higher. While BMI, SBP, DBP, GLU, Hcy, ALT, γ-GT, TBIL, TG, LDL-c, Urea, Cr, UA are higher and HDL-C are lower when men compared to women. This may be related to multiple factors such as genetics, lifestyle and eating habits. There is no significant difference in ALP between the two groups, which may be related to the older average age of the subjects we included. Folic acid, vitamin B12, estrogen, etc. in the human body can promote the metabolism of Hcy. Generally, the serum level of Hcy in women is lower than that in men [11–14]. In this study, the average concentration of Hcy was 10.2 (8.3–12.8) μmol/L, and males were much higher than females. In addition, smoking can indirectly lead to the reduction or lack of folic acid and vitamin B12 levels in the blood and affect the decomposition and metabolism of Hcy [15]. This may also be the reason why the level of Hcy in men is higher than that in women.

From other studies it was found high Hcy related to renal damage [16]. In addition, high UA enhances oxidation and accelerates the production of oxygen free radicals through oxidative stress response, which is ultimately associated with the occurrence and development of cardiovascular and cerebrovascular diseases such as hypertension, coronary atherosclerosis, heart failure and stroke. Many researchers regard UA as an independent risk factor for coronary heart disease. Therefore, in this study, we evaluated Cr and UA as basic data and found that Cr and UA in the high-Hcy group were significantly higher than those in the low-Hcy group. Univariate and multivariate analysis of Hcy normal group and abnormal group showed that Hcy was positively correlated with Cr and UA. In the follow-up study, we will follow up the study subjects to further clarify the relationship between Hcy and kidney injury and other related diseases.

TC, TG, HDL-c, LDL-c are closely related to the occurrence and development of cardiovascular and cerebrovascular diseases. The results of this study showed that the Hcy of abnormal TG and HDL is higher than that of the normal blood lipid group, which is consistent with related literature reports [14, 17]. Some scholars have observed in animal experiments that Hcy injection causes hyperhcy hyperemia, and atherosclerosis occurs 2–3 months later. High levels of Hcy hyperemia was considered to be an independent risk factor for atherosclerosis. Studies have shown that elevated levels of mild and moderate Hcy can increase the risk of death from cardiovascular diseases by 4–6 times, and the risk of CHD increases by 60% in men and 80% in women for every 5 μmol/L of total plasma Hcy level.

High Hcy can damage blood vessel walls and affect lipid metabolism. In this study, both the univariate and multivariate linear regression model analysis results of Hcy in the normal group showed that Hcy was negatively correlated with HDL-c and positively correlated with TG; while in the high Hcy group, Hcy and HDL-c were still negatively correlated with TC. There is a negative correlation. The existing literature reports that high Hcy is negatively related to HDL-c, but the correlation between TC and TG is not consistent in the literature [17]. This may be related to the source of the research object, the geographical distribution, the degree of fasting before sample collection, the number of samples included in the study, and the factors used for correction in the multivariate analysis.

Hcy is a sulfur-containing amino acid produced during the metabolism of methionine in the body. Its main physiological function is to provide methyl groups for many important physiologically active substances such as DNA, protein and phospholipids in the body. Under normal circumstances, the production and metabolism of Hcy in the body maintain a dynamic balance [18], so that the concentration of Hcy is maintained at 5–15 μmol/L in the blood. There are many factors that affect the level of Hcy. In addition, under certain pathological conditions, taking drugs that interfere with metabolism can affect the metabolism of Hcy. The superoxide and peroxide produced can cause vascular endothelial cell damage and vascular smooth muscle cell proliferation. The structural damage of the wall and the increase of lipid deposits in the blood vessel wall accelerate the process of atherosclerosis. Hcy can also destroy the normal coagulation mechanism, increase the chance of thrombosis, and easily increase the risk of arteriosclerotic diseases such as stroke, coronary heart disease, and peripheral vascular disease. Studies have pointed out that for every 5 µmol/L increase in blood Hcy, the risk of ischemic heart disease increases by 32%, and every 3 µmol/L decrease in Hcy, the risk of disease is reduced by 16% [19]. A large number of studies have shown that hyperhomocystaenemia is closely related to the occurrence, development and prognosis of a variety of cardiovascular and cerebrovascular diseases, hypertension, diabetes, and kidney diseases [20–22].

There are still some shortcomings in this study. For example, the fasting state of the study subjects may not be completely consistent, and the liver function is not judged in conjunction with imaging, so detailed evaluation was not performed.

## Conclusion

This study shows that Hcy may participate in or affect the metabolism of HDL-c, Cr, UA, etc. The content of Hcy should be paid attention to in clinical work to provide data support for clinical monitoring of cardiovascular and cerebrovascular diseases and renal function.

## Data Availability

The datasets used and/or analyzed during the current study are de-identifed and available from the corresponding author on reasonable request. Identifying/confidential patient data should not be shared.

## Abbreviations

Hcy: Homocysteine
TC: Total cholesterol
TG: Triglycerides
LDL-c: Low-density lipoprotein cholesterol
HDL-c: High-density lipoprotein cholesterol
ALT: Alanine aminotransferase
ALP: Alkaline phosphatase
γ-GT: γ-Glutamyltransferase
TBIL: Total bilirubin
GLU: Blood glucose
Cr: Creatinine
UA: Uric acid
BMI: Body mass index
SBP: Systolic blood pressure
DBP: Diastolic blood pressure
